# Machine learning of microvolt-level 12-lead electrocardiogram can help distinguish takotsubo syndrome and acute anterior myocardial infarction

**DOI:** 10.1016/j.cvdhj.2022.07.001

**Published:** 2022-07-16

**Authors:** Masato Shimizu, Makoto Suzuki, Hiroyuki Fujii, Shigeki Kimura, Mitsuhiro Nishizaki, Tetsuo Sasano

**Affiliations:** ∗ Department of Cardiology, Yokohama Minami Kyosai Hospital, Yokohama, Japan; † Odawara Cardiovascular Hospital, Odawara, Japan; ‡ Department of Cardiovascular Medicine, Tokyo Medical and Dental University, Tokyo, Japan

**Keywords:** Takotsubo syndrome, Acute anterior myocardial infarction, Machine learning, Electrocardiogram, SHAP method

## Abstract

**Background:**

Qualitative differences in 12-lead electrocardiograms (ECG) at onset have been reported in patients with takotsubo syndrome (TTS) and acute anterior myocardial infarction (Ant-AMI). We aimed to distinguish these diseases by machine learning (ML) approach of microvolt-level quantitative measurements.

**Methods:**

We enrolled 56 consecutive patients with sinus rhythm TTS (median age, 77 years; 16 men), and 1-to-1 random matching was performed based on age and sex of the patients. The ECG in the emergency room was evaluated using an automated system (ECAPs12c; Nihon-Koden). Statistical and ML predictive models for TTS were constructed using clinical features and ECG parameters.

**Results:**

Statistically significant differences were observed in 25 parameters; the V_1_ ST level at the J point (V_1_ STJ) showed the lowest *P* value (*P* < .001). V_1_ STJ ≤+18 μV showed the highest accuracy for TTS (0.773). The highest area under the receiver operating characteristic curve (AUROC) was shown in the aVR ST level at 1/16th of the preceding R-R interval after the J point (aVR STmid: 0.727). Conversely, the light gradient boosting machine (model_LGBM) and extra tree classifier (model_ET) indicated higher accuracy (model_LGBM: 0.842, model_ET: 0.831) and AUROC (model_LGBM: 0.868, model_ET 0.896) than other statistical models. V_1_ STJ had high feature importance and Shapley additive explanation values in the 2 ML models.

**Conclusion:**

ML applied to automated microvolt-level ECG measurements showed the possibility of distinguishing between TTS and Ant-AMI, which may be a clinically useful ECG-based discriminator.

**Key Findings**

• Takotsubo syndrome (TTS) and acute anterior myocardial infarction (Ant-AMI) show similar clinical features, especially in electrocardiogram (ECG) at onset.
• Automated ECG measurement provides microvolt-level ST change; at J point (STJ), at 1/16th of the preceding R-R interval after the J point (STmid), and at 2/16th (STend) in each lead. STJ ≤+18 μV showed the highest accuracy, and aVR STmid indicated the highest area under receiver operating characteristics curve (AUROC) for TTS.
• Diagnostic performance of machine learning (light gradient boosting machine, extra tree classifier) on table data of ECG parameters demonstrated higher accuracy and AUROC for TTS than those of statistical models, and V_1_ STJ played an essential role in building both models.

## Introduction

Takotsubo syndrome (TTS) and acute anterior myocardial infarction (Ant-AMI) at its onset show seemingly similar clinical features, and distinguishing between the 2 diseases without emergent cardiac catheterization is difficult.^1^ Although TTS, a diagnosis of exclusion, can be managed noninvasively with appropriate medical therapy, Ant-AMI is often managed invasively, with ST-elevation myocardial infarction (STEMI) cases requiring emergent revascularization. In cases of non-ST-elevation AMI (NSTEMI) without key clinical symptoms and signs of ST-elevation AMI (STEMI), noninvasive methods are desirable, especially in ambulances, clinics, and hospitals that cannot perform emergent cardiac catheterization. STEMI cases should be transferred to hospitals with cardiac catheterization laboratories immediately, but even in such hospitals, emergent catheterization is sometimes difficult to perform owing to various reasons: advanced age, dementia, frailty, poor physical status, and/or social problems. Twelve-lead electrocardiogram (ECG) is a fundamental examination that can be performed on arrival and has a time advantage compared to other examinations such as high-sensitivity troponin. Initial ECG on arrival can be useful to triage patients, but ECG between the 2 diseases at onset shows very similar patterns. The difference in ECG has been studied by several investigators.^2^^,^^3^ Difference in ST-T change has also been well studied, and several leads (eg, V_1_, aVR, inferior leads) were reported to have important roles in distinguishing between the diseases.^2^^,^^3^ Although these reports demonstrated good accuracy (0.95, Kosuge and Kimura,^2^ and 0.66-0.86, Jim and colleagues),^3^ external validity was not confirmed.

Machine learning (ML) applied to ECG has been developed in several cardiac diseases, and some investigators have reported methods for diagnosing myocardial infarction using a convolutional neural network on vector data of ECG.^4^^,^^5^ However, the accuracy (0.81, Makimoto and colleagues^4^) or area under the receiver operating characteristic curve (AUROC; 0.85–0.88, Cho and colleagues^5^) was not higher than that of conventional ST-level examination. Moreover, there are no studies on the diagnosis of TTS using ML applied to ECG.

We aimed to build a predictive model for TTS by ML, not with ECG vector data but with table data of conventional 12-lead ECG parameters, and to elucidate the parameters of ECG with high feature importance in the ML models.

## Methods

### Study patients and ECG

We enrolled 56 consecutive patients at Yokohama Minami Kyosai Hospital with sinus rhythm with apical ballooning–type TTS from 2013 to 2021. In all cases, cardiac catheterization was performed, and no coronary stenosis and left ventricular apical ballooning was confirmed in TTS cases. Stenosis/occlusion of the left anterior descending coronary artery was confirmed in the Ant-AMI cases. The diagnosis of TTS was based on Mayo’s criteria,^1^ and other diseases that mimicked AMI (acute myocarditis/pericarditis and vasospastic angina) were excluded by absence of inflammation or acetylcholine provocation test (several days after admission). The diagnosis of AMI (STEMI and NSTEMI) was based on the fourth universal definition of AMI.^6^ Among our AMI database, patients with same age and sex in each TTS case were extracted, and 1-to-1 random matching was performed. Finally, 112 patients (median age, 77 years [interquartile range, 67–84 years]; 32 men) were enrolled.

**Figure 1:**
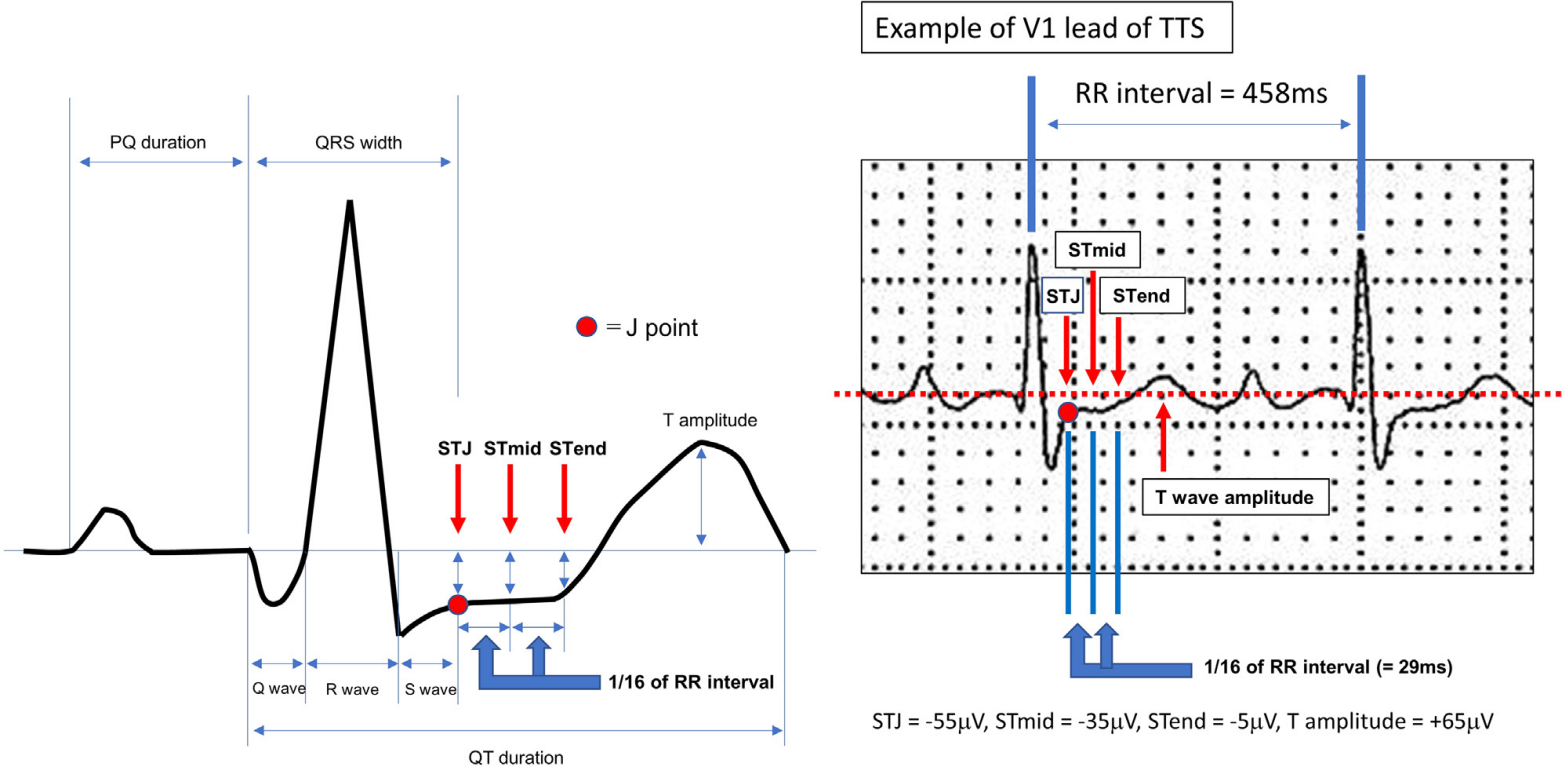
Explanation of measurement on 1 beat of the electrocardiogram (ECG). All the parameters were measured automatically. Left figure shows schema of the measurement, and right figure a real ECG wave and real results. The ST level was measured at 3 points: (1) ST level at the J point (STJ), which was recorded at the end of the QRS complex as measured in μV with respect to the baseline; (2) the middle of the ST level (STmid), which is the ST level at the point of 1/16^th^ of the preceding R-R interval after the J point; and (3) the end of the ST level (STend), which is the ST level at the point of 2/16^th^ of the preceding R-R interval after the J point. The T-wave amplitude was defined as the absolute distance from the apex of the T wave to the baseline. TTS = takotsubo syndrome.

ECG on arrival (in the emergency room) in both groups was measured at the μV level using an automated system (ECAPs12c; Nihon-Koden, Tokyo, Japan).^7^ ECG variables, ST levels, T-wave amplitude, and other fundamental parameters were preselected, as explained in Figure 1, and those parameters were measured from 10-second waveforms. The ST levels of each lead were measured automatically at 3 points: (1) ST level at the J point (STJ), which was recorded at the end of the QRS complex, measured with respect to the baseline; (2) the middle of the ST level (STmid), which is the ST level at the point 1/16^th^ of the preceding R-R interval after the J point; and (3) the end of the ST level (STend), at the point 2/16^th^ of the preceding R-R interval after the J point. Qualitative ST elevation/depression was defined as at least 0.1 μV deviation at J point, judged on the automated measurement. The T-wave amplitude was defined as the absolute distance from the apex of the T wave to the baseline. The rate-corrected QT interval (QTc) was calculated using the modified Framingham (ECAPs12C) formula (QTc = QT + [1000 – R-R]/7).

The ethics committee of Yokohama Minami Kyosai Hospital approved the study protocol and written informed consent was obtained from all participants prior to the study.

### Statistical analysis for characteristics of patients

Numeric variables are displayed as the median value (interquartile range: 25%–75% value), and the Mann–Whitney test was used to compare the TTS and Ant-AMI groups. Fisher exact test was used to evaluate differences in categorical variables, and Holm’s multiple comparison was used as a post hoc test.

Statistical significance was set at *P* < .05. All statistical analyses were performed using EZR (Saitama Medical Center, Jichi Medical University, Saitama, Japan),^8^ a graphical user interface for R (The R Foundation for Statistical Computing, Vienna, Austria).^9^

### Predictive model construction by statistical method

Fifty-six pairs of cases were randomly split into 80% and 20% (45 pairs vs 11 pairs), in which 45 pairs (90 cases) were set as the prediction data and 11 pairs (22 cases) as the test data. Statistical predictive models were constructed based on these 90 cases, as described below. Univariate logistic regression analysis for TTS was performed using the prediction data, and significant predictors were extracted. A multivariate logistic regression analysis was not performed because of the multicollinearity of many pairs of parameters. A receiver operating characteristic (ROC) curve analysis was performed, and the cutoff value was calculated based on the Youden index. The statistical predictive model consisted of an assessment of whether a parameter in each case was higher/lower than the cutoff value (named the cutoff value model). A confusion matrix was created, and the diagnostic performance (accuracy/sensitivity : recall/positive predictive value : precision) was evaluated. From the analysis of the predictive model, the propensity score (PS) of each predictor was calculated, and the PS formula for each predictor was constructed (the ROC curve model):

PS formula=1/(1+exp(−αx+β))

where α = coefficient of predictor and β = intercept, calculated by logistic regression analysis. The model was applied to the test data and the AUROC was measured using ROC curve analysis.

### Predictive model construction and validation by ML

Among the ML methods for ECG data, we did not use conventional deep learning procedures using 1-dimensional data (vector data)^10^ because it was difficult to explain the feature importance in the model. To secure explainability, we adopted a novel method that used an ensemble learning procedure with conventional ECG parameters (eg, ST level, T-wave amplitude) as table data.

Eleven ML models were built using PyCaret, an open-source wrapper over several ML libraries in Python in a low-code environment.^11^ After screening of the 11 models, we adopted 2 excellent ensemble learning methods: an extra tree classifier (model_ET) and a light gradient boosting machine (model_LGBM),^12^^,^^13^ which uses many random decision trees and built a majority vote-like system. A brief explanation of the ensemble learning is provided in Supplemental File 1. All 112 cases were randomly split 10 times into 80% of the data for ML training (90 cases) and 20% of the data for validation (22 cases), and 10-fold random cross-validation was performed. Both models were tuned to obtain the highest accuracy with optimization of the hyperparameters. The best number of features was searched by recursive feature elimination by cross-validation on PyCaret. The cross-validated model was finalized on PyCaret (the results of hyperparameter tuning are displayed in Supplemental File 6).

The feature importance of the models was ranked to estimate the contribution of the predictors to the ML models. In addition to feature importance, the SHAP (SHapley Additive exPlanations) method was introduced on data for ML model training.^14^^,^^15^ The theory of SHAP is based on “the game optimal Shapley values,”^14^ and the summary plot of SHAP combines feature importance with feature effects. The red and blue points indicate TTS and Ant-AMI, respectively. On the x-axis, the Shapley value of each feature is displayed (defined as the SHAP value), in which a large (right side) value corresponds to a positive contribution to the model. A brief explanation of the SHAP method is provided in Supplemental File 2.

## Results

The characteristics of all 112 patients and the comparison of prediction data (n = 90) and test data (n = 22) are displayed in Supplemental File 2. There were no significant differences between both the groups.

Table 1 shows the qualitative ST elevation/depression in V_1_–V_4_ leads and the number of cases diagnosed as anterior STEMI. Although there were significant differences in V_1_ ST elevation (*P* < .001) and V_4_ ST depression (*P* = .029) at point J, the number of cases diagnosed with anterior STEMI did not differ between the 2 diseases (TTS, 28; Ant-AMI, 31; *P* = .566).

**Table 1:** Comparison of qualitative ST elevation/depression and T-wave inversion in takotsubo syndrome and acute anterior myocardial infarction

		All cases (n = 112)				Prediction data (n = 90)	
		TTS (n = 56)	AMI (n = 56)	*P* value	*P* value (post hoc)	TTS (n = 45)	AMI (n = 45)
Anterior STEMI		28	31	.566		23	26
V_1_	ST elevation at J point	3	18	<.001	<.001 (ST elevation)	2	14
	ST depression at J point	0	0			0	0
	T-wave inversion	7	9	.793		5	7
V_2_	ST elevation at J point	27	33	.462		22	28
	ST depression at J point	1	2			0	1
	T-wave inversion	8	5	.557		6	4
V_3_	ST elevation at J point	28	28	.302		24	11
	ST depression at J point	28	25			0	1
	T-wave inversion	11	10	1.000		8	7
V_4_	ST elevation at J point	23	17	.029	.032 (ST depression)	18	14
	ST depression at J point	2	11			1	7
	T-wave inversion	15	16	1.000		11	13

ST elevation/depression was defined as at least 0.1 μV ST deviation at J point, and T-wave inversion as at least -0.1 μV amplitude judged on automated measurement. Diagnosis of ST-elevated acute anterior myocardial infarction was based on fourth universal definition of acute myocardial infarction—briefly, ST elevation at the J-point in 2 contiguous leads with the cut point ≥1 mm in V_1_–V_4_ leads, or other than leads V_2_–V_3_ where the following cut-points apply: ≥2 mm in men ≥40 years; ≥2.5 mm in men <40 years, or ≥1.5 mm in women regardless of age. Fisher exact test was performed, and as post hoc analysis, data underwent Holm’s multiple comparison. A *P* < .05 was considered as significant.

Table 2 shows a comparison of the TTS and Ant-AMI in the prediction data (n = 90). Hyperlipidemia and diabetes cases with TTS were significantly lower than those with Ant-AMI. Among ECG parameters, heart rate and several ST levels (lead I/II/aVR/aVF/V_1_/V_2_/V_5_/V_6_) demonstrated significant differences. Univariate logistic regression analysis identified 25 significant predictors (asterisk [∗] in Table 2). Multivariate logistic regression analysis was not performed because of the many significant correlations/confounding/multicollinearity among the variables.

**Table 2:** Comparison of takotsubo syndrome, acute anterior myocardial infarction, and univariate logistic regression analysis for takotsubo syndrome, on prediction data (n = 90)

	Comparison of TTS and Ant-AMI		Univariate logistic regression for TTS		
	TTS (n = 45)	Ant-AMI (n = 45)	OR	95% CI	*P* value
Age (years)	78 [69, 86]		NA		
Male	14 (31%)		NA		
HTN	14 (31%)	19 (42%)	0.618	0.26-1.47	.275
HL	9 (20%)	29 (64%)	0.138	0.05-0.36	<.001∗
DM	2 (4%)	14 (31%)	0.103	0.02-0.49	.004∗
CKD	14 (31%)	8 (18%)	2.090	0.78-5.63	.145
BNP (pg/mL)	274 [67, 482]	184 [59, 477]	0.999	0.94-1.07	.982
WBC (/mm^3^)	8200 [6700, 11325]	9200 [7100, 11400]	1.020	0.92-1.13	.723
CRP (mg/dL)	1.38 [0.23, 6.58]	0.23 [0.10, 0.76]	1.100	1.00-1.21	.053
HR (bpm)	95 [79, 131]	84 [71, 94]	1.030	1.01-1.05	.002∗
P axis (degree)	57 [38, 73]	55 [40, 62]	1.000	0.99-1.02	.472
PR (ms)	168 [154, 187]	172 [156, 188]	0.996	0.98-1.01	.587
QRS axis (degree)	35 [-19, 74]	22 [-2, 49]	1.000	1.00-1.01	.345
QRS width (ms)	90 [84, 100]	92 [82, 102]	0.976	0.76-1.25	.849
QTc (ms)	429 [410, 449]	430 [405, 442]	1.030	0.92-1.15	.611
T axis (degree)	67 [37, 94]	69 [20, 106]	1.000	1.00-1.01	.296
I STJ	20 [-40, 15]	-15 [-45, 15]	1.160	1.05-1.28	.003∗
I STmid	10 [-5, 55]	-10 [-40, 30]	1.100	1.02-1.19	.015∗
I STend	30 [-5, 60]	-5 [-35, 50]	1.040	0.99-1.10	.164
I T	68 [-36, 156]	58 [-53, 146]	1.080	0.82-1.42	.571
II STJ	20 [-10, 75]	-20 [-55, 30]	1.120	1.04-1.20	.002∗
II STmid	25 [0, 85]	0 [-55, 35]	1.120	1.05-1.20	.001∗
II STend	50 [5, 105]	10 [-40, 65]	1.080	1.02-1.14	.005∗
II T	145 [90, 265]	160 [63, 278]	1.000	0.79-1.27	.971
III STJ	5 [-35, 35]	5 [-60, 60]	1.030	0.97-1.08	.366
III STmid	5 [-25, 50]	0 [-65, 60]	1.040	0.99-1.09	.155
III STend	15 [-25, 55]	25 [-60, 70]	1.030	0.99-1.07	.154
III T	100 [-45, 195]	108 [-93, 203]	1.040	0.95-1.29	.678
aVR STJ	-20 [-60, 3]	10 [-15, 40]	0.830	0.74-0.93	<.001∗
aVR STmid	-30 [-70, -5]	5 [-25, 35]	0.831	0.75-0.92	<.001∗
aVR STend	-40 [-75, -15]	0 [-50, 30]	0.902	0.94-0.97	.006∗
aVR T	-120 [-185, -65]	-115 [-195, -50]	0.966	0.70-1.33	.831
aVL STJ	13 [-15, 46]	-15 [-40, 35]	1.060	0.98-1.15	.144
aVL STmid	5 [-15, 30]	-5 [-40, 25]	1.010	0.95-1.08	.796
aVL STend	0 [-15, 35]	-5 [-50, 45]	0.993	0.95-1.04	.784
aVL T	35 [-78, 78]	-33 [-119, 123]	1.040	0.80-1.36	.754
aVF STJ	10 [-20, 55]	-10 [-60, 45]	1.080	1.01-1.15	.027∗
aVF STmid	10 [-10, 60]	0 [-70, 50]	1.090	1.02-1.16	.011∗
aVF STend	20 [-5, 75]	5 [-60, 70]	1.060	1.01-1.12	.026∗
aVF T	135 [75, 225]	125 [-8, 220]	0.976	0.94-1.01	.155
V_1_ STJ	15 [-20, 35]	70 [20, 130]	0.846	0.78-0.92	<.001∗
V_1_ STmid	30 [-10, 60]	100 [40, 185]	0.860	0.80-0.93	<.001∗
V_1_ STend	40 [-15, 65]	120 [30, 220]	0.917	0.87-0.97	.001∗
V_1_ T	50 [-70, 185]	143 [55, 289]	0.786	0.62-0.99	.044∗
V_2_ STJ	93 [31, 138]	135 [45, 290]	0.959	0.93-0.99	.018∗
V_2_ STmid	135 [75, 205]	215 [100, 420]	0.960	0.93-0.99	.005∗
V_2_ STend	185 [80, 280]	295 [130, 550]	0.981	0.96-1.00	.031∗
V_2_ T	300 [150, 490]	435 [235, 815]	0.932	0.94-1.03	.167
V_3_ STJ	110 [45, 170]	65 [-30, 290]	1.000	0.98-1.03	.777
V_3_ STmid	170 [70, 240]	190 [30, 390]	0.995	0.98-1.02	.63
V_3_ STend	215 [105, 320]	290 [25, 500]	0.991	0.98-1.01	.251
V_3_ T	330 [80, 540]	435 [95, 725]	0.960	0.88-1.05	.363
V_4_ STJ	70 [22, 150]	0 [-55, 140]	1.020	0.99-1.04	.205
V_4_ STmid	105 [40, 185]	55 [-15, 230]	1.010	0.99-1.03	.623
V_4_ STend	160 [45, 255]	140 [-10, 295]	1.000	0.98-1.02	.987
V_4_ T	263 [-89, 493]	205 [-130, 505]	0.985	0.90-1.08	.739
V_5_ STJ	50 [15, 100]	-20 [-60, 45]	1.050	1.01-1.09	.015∗
V_5_ STmid	70 [15, 125]	-5 [-55, 85]	1.030	1.00-1.06	.073
V_5_ STend	75 [0, 155]	-10 [-55, 130]	1.010	0.99-1.03	.337
V_5_ T	135 [-105, 275]	95 [-155, 260]	1.010	0.89-1.13	.922
V_6_ STJ	25 [-5, 55]	-35 [-65, 10]	1.150	1.06-1.25	<.001∗
V_6_ STmid	25 [-5, 70]	-25 [-60, 5]	1.110	1.04-1.18	.002∗
V_6_ STend	40 [-10, 100]	-25 [-65, 40]	1.060	1.01-1.11	.010∗
V_6_ T	93 [41, 195]	65 [-135, 233]	1.060	0.88-1.26	.556

Numeric variables are displayed as median [interquartile range: 25%, 75%], and categorical variables are displayed as n (%). STJ, STmid, STend, and T wave are expressed as μV, and are explained in Figure 1. CK and CKMB were maximum value during acute phase, and they were not analyzed by logistic regression. Statistical comparison methods, abbreviations are explained in Table 1 footnote. In logistic regression, BNP were analyzed per 100 pg/mL, and the result of OR and 95% CI were displayed as per 100 values. WBC per 100 counts/mm^3^, ST levels per 10 μV, and T-wave amplitude per 100 μV. *P* < .05 was considered significant; significant values are denoted by an asterisk (∗).

Ant-AMI = acute anterior myocardial infarction; BNP = brain natriuretic peptide; bpm = beats per minute; CI = confidence interval; CKD = chronic kidney disease; CRP = C-reactive protein; DM = diabetes mellitus; HL = hyperlipidemia; HR = heart rate; HTN = hypertension; N/A = not applicable; OR = odds ratio; TTS = takotsubo syndrome; WBC = white blood cell.

The diagnostic performances of the statistical predictive models are presented in Table 3, and the ML models in Table 4. Among the statistical predictors, V_1_ STJ ≤18 μV showed the highest accuracy of 0.773 (in test data), and aVR STmid had the highest AUROC (0.727, in test data). The results of recursive feature elimination by cross-validation are demonstrated in Supplemental File 4. In model_LGBM, the best number of features was 16, 24, and 25; and in model_ET, 25. As a result, we adopted all 25 features to construct the ML models. Compared with the statistical predictive models, model_LGBM and model_ET had higher accuracy (0.842 and 0.831, respectively) and AUROC (0.868 and 0.896, respectively).

**Table 3:** Diagnostic performance of statistical predictive models

	Prediction data (n = 90)						Test data (n = 22)					
	ROC curve model		Cutoff value model				ROC curve model		Cutoff value model			
	Cutoff	AUROC	Acc	Recall (Sens)	Prec. (PPV)	F1	AUROC	95% CI of AUROC	Acc	Recall (Sens)	Prec. (PPV)	F1
HL			0.278	0.200	0.237	0.217			0.318	0.364	0.333	0.348
DM (n, %)			0.367	0.044	0.125	0.066			0.227	0.091	0.125	0.105
HR (bpm)	≥109	0.665	0.678	0.422	0.864	0.567	0.554	0.30-0.81	0.455	0.091	0.333	0.143
I STJ	≥+5 μV	0.711	0.711	0.733	0.702	0.717	0.649	0.39-0.91	0.591	0.545	0.600	0.571
I STmid	≥-5 μV	0.681	0.689	0.800	0.655	0.720	0.665	0.42-0.91	0.591	0.545	0.600	0.571
II STJ	≥-20 μV	0.672	0.644	0.800	0.610	0.692	0.674	0.58-0.77	0.636	0.909	0.588	0.714
II STmid	≥+10 μV	0.701	0.678	0.733	0.660	0.695	0.661	0.42-0.90	0.545	0.636	0.538	0.583
II STend	≥-5 μV	0.667	0.644	0.867	0.600	0.709	0.624	0.38-0.87	0.636	0.909	0.588	0.714
aVR STJ	≤-20 μV	0.729	0.693	0.605	0.722	0.658	0.698	0.46-0.94	0.591	0.545	0.600	0.571
aVR STmid	≤-10 μV	0.732	0.678	0.711	0.667	0.688	0.727	0.51-0.95	0.636	0.636	0.636	0.636
aVR STend	≤0 μV	0.693	0.667	0.867	0.619	0.722	0.694	0.47-0.92	0.591	0.636	0.583	0.609
aVF STJ	≥-40 μV	0.615	0.633	0.911	0.586	0.713	0.636	0.40-0.88	0.545	0.909	0.526	0.667
aVF STmid	≥-35 μV	0.635	0.656	0.956	0.597	0.735	0.579	0.33-0.83	0.545	1.000	0.524	0.688
aVF STend	≥-30 μV	0.614	0.633	0.911	0.586	0.713	0.463	0.20-0.73	0.500	0.909	0.500	0.645
V1 STJ	≤+18 μV	0.771	0.611	0.267	0.857	0.407	0.690	0.46-0.92	0.773	0.636	0.875	0.737
V1 STmid	≤+120 μV	0.765	0.700	0.956	0.632	0.761	0.653	0.40-0.90	0.645	1.000	0.524	0.688
V1 STend	≤+85 μV	0.699	0.667	0.778	0.636	0.700	0.628	0.37-0.89	0.682	1.000	0.611	0.759
V1 T	≤+50 μV	0.635	0.663	0.538	0.677	0.600	0.645	0.38-0.92	0.667	0.600	0.667	0.632
V2 STJ	≤+130 μV	0.629	0.652	0.750	0.623	0.680	0.616	0.37-0.87	0.636	0.818	0.600	0.692
V2 STmid	≤+205 μV	0.658	0.656	0.756	0.630	0.687	0.665	0.56-0.77	0.682	0.909	0.625	0.741
V2 STend	≤+280 μV	0.635	0.633	0.756	0.607	0.673	0.661	0.42-0.90	0.682	0.909	0.625	0.741
V5 STJ	≥+5 μV	0.724	0.733	0.822	0.698	0.755	0.645	0.40-0.89	0.591	0.636	0.583	0.609
V6 STJ	≥-5 μV	0.768	0.733	0.800	0.706	0.750	0.669	0.43-0.91	0.682	0.818	0.643	0.720
V6 STmid	≥-10 μV	0.749	0.733	0.822	0.698	0.755	0.674	0.43-0.92	0.682	0.909	0.625	0.741
V6 STend	≥-30 μV	0.714	0.700	0.933	0.636	0.757	0.636	0.39-0.89	0.682	0.909	0.625	0.741

Initially, a receiver operating characteristic (ROC) curve analysis was performed, area under ROC (AUROC) was measured, and cutoff value was calculated by Youden index. AUROC of hyperlipidemia (HL) and diabetes (DM) were not evaluated because they were bivariate categorical variables. The statistical predictive model consisted of 2 methods, an assessment of whether a parameter of each case had higher/lower value than the cutoff (named as cutoff value model), and propensity score (PS) of each predictor was calculated on the prediction data, and the PS formula for each predictor was constructed (named as ROC curve model), which was applied to the test data and AUROC was measured by ROC curve analysis. Confusion matrix was prepared from the model, and diagnostic performance (accuracy [Acc] / sensitivity [Sens]; named as recall / positive predictive value [PPV]; named as precision [Prec.] / F1 score [harmonic mean of recall and prec.]) was calculated. Abbreviations are explained in Table 2 footnote.

**Table 4:** Results of validation data of machine learning predictive models, which were built by PyCaret

	Acc	AUROC	Recall (Sens)	Prec. (PPV)	F1
Light gradient boosting machine	0.856	0.865	0.870	0.867	0.860
Extra trees classifier	0.832	0.881	0.830	0.863	0.836
Ada boost classifier	0.832	0.874	0.875	0.825	0.840
Naive Bayes	0.821	0.874	0.810	0.867	0.822
Gradient boosting classifier	0.821	0.865	0.870	0.835	0.837
Random forest classifier	0.821	0.850	0.850	0.848	0.831
Linear discriminant analysis	0.786	0.844	0.805	0.808	0.788
Decision tree classifier	0.778	0.810	0.825	0.778	0.783
K neighbors classifier	0.776	0.794	0.850	0.777	0.797
Logistic regression	0.719	0.799	0.715	0.775	0.717
Quadratic discriminant analysis	0.708	0.688	0.790	0.729	0.734

The diagnostic performance was explained by accuracy (Acc) / sensitivity (Sens); named as recall / positive predictive value (PPV); named as precision (Prec.) / and F1 score (harmonic mean of recall and Prec.). Ten times random cross-validation was performed, and the average of results was displayed.

**Figure 2:**
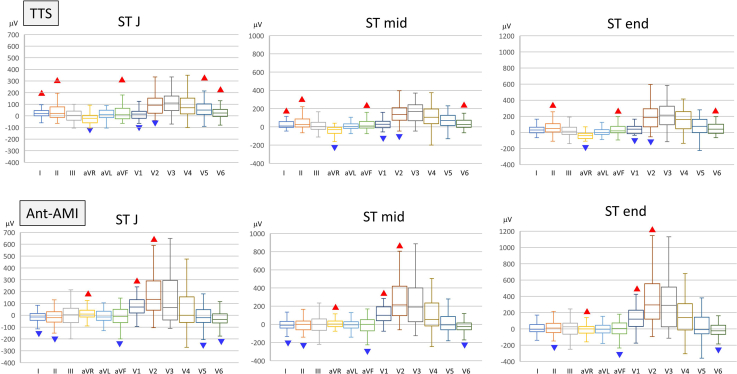
Comparison of ST levels of takotsubo syndrome (TTS) and acute anterior myocardial infarction (Ant-AMI) in prediction data (90 cases). Left figure shows ST level at the J point (STJ), middle figure shows the middle of the ST level (STmid), and right figure shows the end of the ST level (STend). After Mann–Whitney *U* test, a box-and-whisker plot was drawn in each lead. The box indicates interquartile range and median value, and the whisker corresponds to maximum and minimum value. The red and blue triangles show significant upper and lower values, respectively.

**Figure 3:**
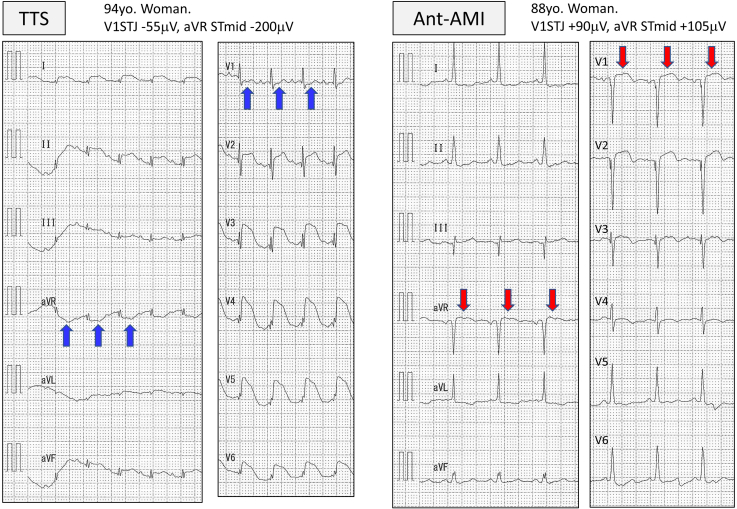
Representative 12-lead electrocardiograms. Left figure demonstrates a takotsubo syndrome (TTS) case, in which ST repression is observed in aVR and V_1_. Right figure displays an ST-elevation acute anterior myocardial infarction (Ant-AMI) case, in which ST elevation is found in aVR and V_1_–V_3_. The patient’s coronary arteriography showed occlusion of the left anterior descending branch (segment 6).

Figure 2 shows a comparison of ST levels. The STJ of TTS in lead I/II/aVF/V_5_/V_6_ was higher than that in Ant-AMI, and the STJ of TTS in lead aVR/V_1_/V_2_ was lower than that in Ant-AMI. The STmid and STend showed similar results to STJ, but V_5_ STmid, V_5_ STend, and I STend were not significant predictors. The representative ECG waveforms are shown in Figure 3. From Table 3, the representative ECG characteristics of TTS compared with Ant-AMI were as follows: no ST elevation of V_1_ STJ (≤+18 μV) and ST depression in aVRmid (≤-10 μV). The representative and visible ECG features of TTS were summarized as no ST elevation in V_1_ and ST depression in aVR.

**Figure 4:**
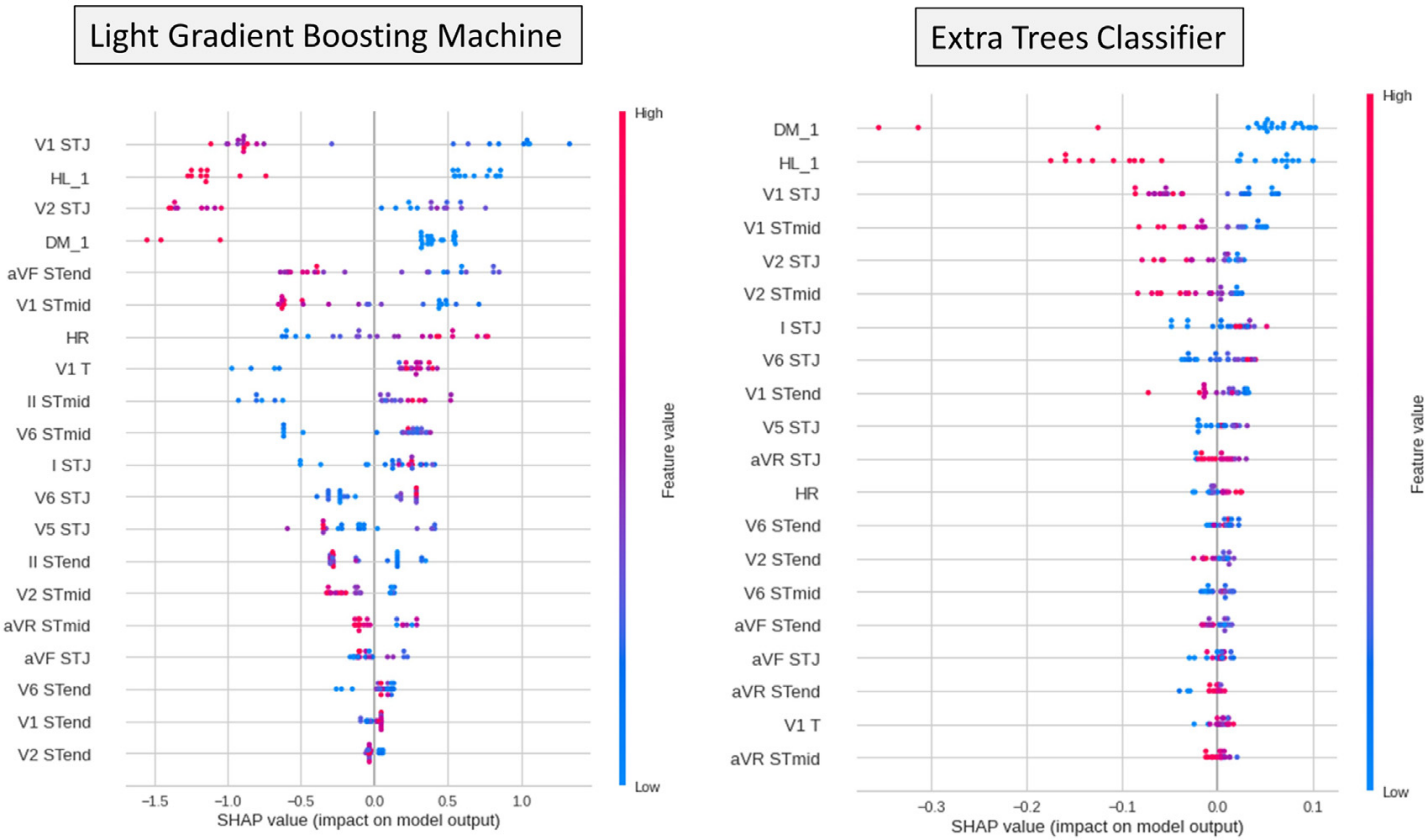
Interpretation of feature importance by SHapley Additive exPlanation (SHAP) method on 2 representative machine learning models. Left side: Plot of light gradient boosting machine; right side: plot of extra trees classifier. Each point on the summary plot corresponds to a SHAP value for a feature and an instance. Each red and blue point shows a case with takotsubo syndrome and acute anterior myocardial infarction, respectively. On the y-axis, features are sorted based on their importance; color shows the feature value from low (*blue*) to high (*red*). The SHAP value is displayed on the x-axis, wherein left side (minus value) shows negative impact and right side (plus value) shows positive impact. Abbreviations as in Figure 1 and Table 2. Brief explanation of SHAP method is demonstrated in Supplemental File 2.

The important features of the 2 ML models are shown in Supplemental File 5. In model_LGBM, the V_1_ STJ showed the highest feature importance. Conversely in model_ET, not only V_1_ STJ but diabetes and hyperlipidemia showed high feature importance. The SHAP values of the models are shown in Figure 4, which showed similar pattern to Supplemental File 5. V_1_ STJ showed the highest feature value in model_LGBM; but in model_ET, V_1_ STJ, hyperlipidemia, and diabetes played important roles in model building.

## Discussion

We built predictive models for TTS and Ant-AMI using an automated ECG system with μV-level measurements. The ST levels in several leads were significant predictors, and we were able to provide clinically useful cutoff values for them, as shown in Table 3. Among them, V1 STJ ≤+18 μV showed the highest accuracy, and aVR STmid showed the highest AUROC in test data. Conversely, ML predictive models demonstrated higher accuracy and AUROC than statistical models. In the ML models, V_1_ STJ played an important role in model building.

### ST level as a predictor of TTS

In addition to ST levels in V_1_, ST levels in I/II/aVR/aVF/V_2_/V_6_ were significant predictors of TTS.

The significance of lead V_2_ can be explained by the importance of the nearest lead, V_1_, which is well known. Kosuge and colleagues^2^ explained that V_1_ is located in both the right ventricular anterior region and the right paraseptal region; however, abnormalities of wall contraction in TTS rarely extend to the right ventricle region, compared with AMI. Therefore, the significance of other leads can be explained as follows:

#### Lead aVR

Several investigators found more ST depression in TTS than in Ant-AMI.^2^^,^^16^^,^^17^ Ant-AMI causes greater injury than TTS. In the present study, the CK-MB level in Ant-AMI was higher than that in TTS. Lead aVR is known as a “cavity lead” and allows for visualization of the left ventricle (LV); therefore, the aVR lead can help determine the total damage of the LV.^18^

#### Lead II/aVF

ST elevation was found in inferior leads in 33%–50% of TTS cases in large series.^19^^,^^20^ Jim and colleagues^3^ emphasized the importance of ST elevation in the inferior leads as a new criterion for TTS diagnosis. They described that the inferior myocardium is universally affected in typical TTS, theoretically expressed as inferior ST-segment elevation. Compared with TTS, Ant-AMI tends to show large LV damage, in which the vector of injuries of opposing walls cancel each other out, and simultaneous ischemia in both the lateral and inferior walls reduces the ST-segment changes in their respective leads.

#### Lead V_6_/I

Inoue and colleagues^21^ reported more prevalent V_6_ ST elevation in TTS than in Ant-AMI. Ogura and colleagues^20^ reported that the ratio ΣST elevation of V_4_–V_6_ / ΣST elevation of V_1_–V_3_ was a significant predictor of TTS. Regarding lead I of TTS, there were no reports. However, these differences can easily be understood from the perspective of RV involvement. Chia and colleagues^22^ reported ST depression of the lateral lead as a sign of right ventricular ischemia. Both lead V_1_ ST elevation and lead I/V_6_ ST depression can show RV involvement of Ant-AMI.

### Machine learning of ECG to distinguish between TTS and Ant-AMI

Although the accuracy was not very high for each of the 25 significant predictors, their aggregation created excellent predictive ML models with the algorithms Model_ET and Model_LGBM, which are ensemble learning models and use decision trees.^12^^,^^13^ The method of model_ET to divide trees is based not on the best fit method but on a random choice of the Gini coefficient or entropy; consequently, model_ET can show high performance, especially in the presence of noisy features.^12^^,^^23^ Therefore, model_ET has an advantage if the importance of each variable is not very high. In the present study, the accuracy of 22 significant predictors was limited, and model_ET was suitable for building a predictive model.

Model_LGBM is frequently used and is known to exhibit higher diagnostic performance, especially on table data, compared to other ensemble ML methods.^12^^,^^24^ Following are the advantages of model_LGBM: (1) Model_LGBM uses the boosting method, which is a series data composition, instead of bagging (bootstrap aggregating; used in random forest method). Consequently, the learning speed is faster than that of the parallel data composition of bagging. (2) Decision trees of model_LGBM are a leaf-wise tree growth method, which is much faster than the level-wise tree growth method (used, eg, in XG boosting). (3) Fine-tuning of hyperparameters can be performed more easily in model_LGBM than in other ML models, which improves the accuracy of the model. Therefore, model_LGBM can perform excellently under the condition of many low-importance parameters, such as in the present study.

The SHAP method is novel and can show both feature importance and correlation (positive or negative). In statistical comparison by multivariate analysis, it is difficult to compare the importance of all parameters when there are pairs with significant correlation/confounding/multicollinearity. However, in ML (especially ensemble learning models using decision trees), most models are adjusted by internal regularization, and their predictive value is not affected. The explainability of features is somewhat affected (weakened) when there is significant correlation, confounding, or multicollinearity; as opposed to statistical models, parameters are recognized not to exclude because of internal regularization.^25^

### Study limitations

This study was performed with a small sample size of patients; therefore, several limitations were inevitable: no external validation (using separate external test data), no ECG variations, and no validation in other similar clinical populations (acute pericarditis, inferior AMI, atypical TTS, no sinus rhythm, etc). The precision of the study became relatively low because of the small sample size of test data. We did not perform deep learning as ML method because of the size. Because STEMI and NSTEMI cases are treated differently, it was ideal to separate the 2 groups. Combining multiple leads seems to produce good results, but in our preliminary data, it induced overfitting to prediction data and we could not show the usefulness of the combination. Although the V_1_ STJ was essential in both ML models, other important features of the models were not the same; hence, the diagnosis by the 2 models might be different in several patients. An automated system of ECG (ECAPs12c) with μV-level measurement demonstrated a higher diagnostic performance; however, this system is not commonly used worldwide.

## Conclusion

ML on the parameters of the automated ECG system with μV measurement showed superior diagnostic performance compared to conventional single ECG parameters to distinguish between TTS and Ant-AMI. Although the results of the present study were limited by the small sample size, it may be a clinically useful ECG-based discriminator.
