# Supplementary material for: Machine learning of microvolt-level 12-lead electrocardiogram can help distinguish takotsubo syndrome and acute anterior myocardial infarction
Source: Cardiovasc Digit Health J. 2022 Jul 16;3(4):179–88. doi: 10.1016/j.cvdhj.2022.07.001 (PMC9422059; doi:10.1016/j.cvdhj.2022.07.001)
Supplement: Supplementary Data [file mmc1.docx]

**Supplemental file 1**

**
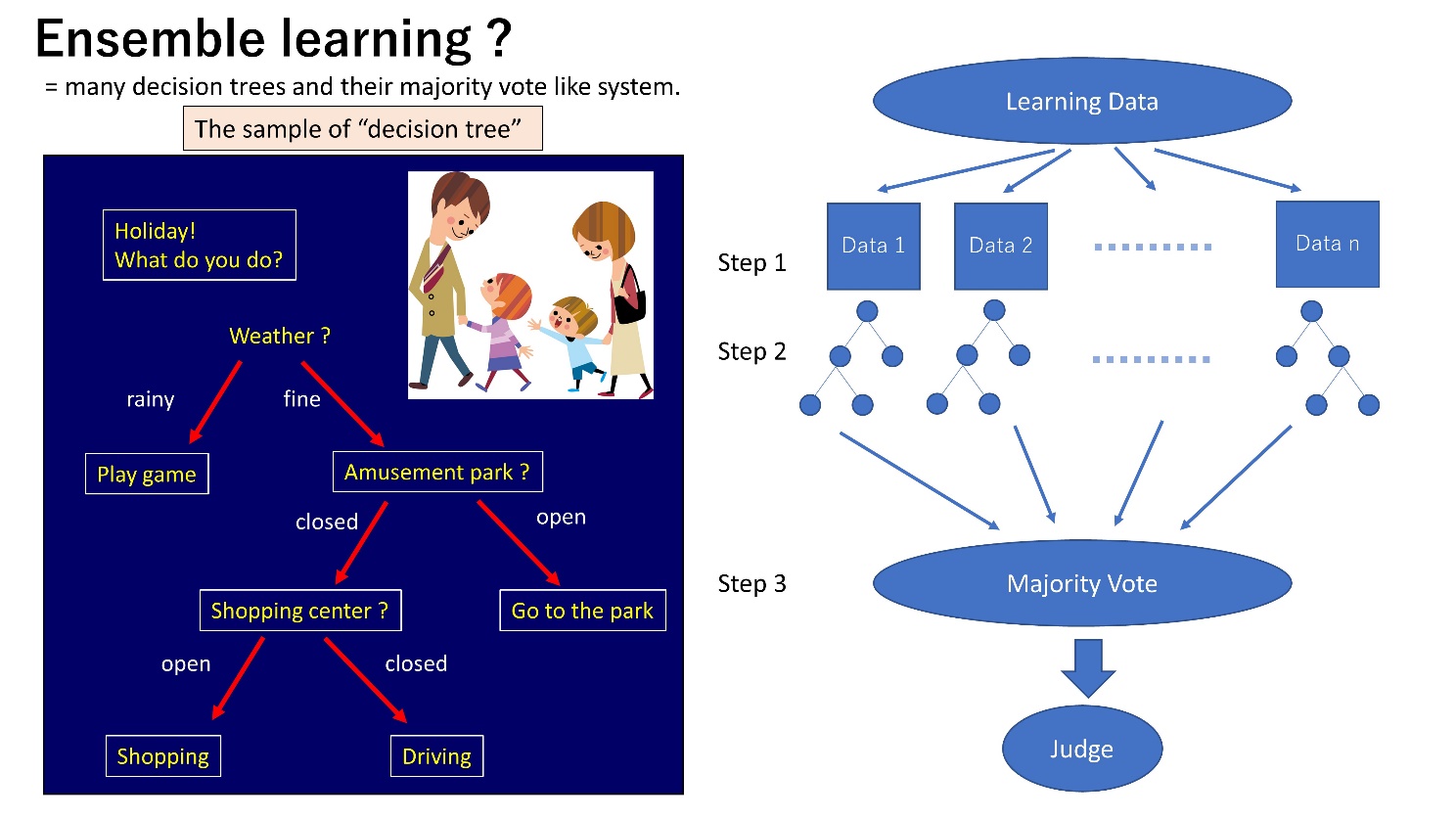
**

Explanation of ensemble learning. Ensemble learning is one of machine learning method, which uses many decision trees and construct their majority voting like system. The left figure explains a sample of decision tree, and right figure an image of majority vote. The image is explained by “the wisdom of the crowd”, which indicates that many people with average knowledge are able to answer to a problem. The aggregation of the answers can cancel out the noise, and as the results, it goes beyond highly knowledgeable experts. The same rule can apply to machine learning which is based on mathematical models.

Illustration by Enosuke (<https://pixta.jp>, material number 55346096).

**Supplemental file 2**

**
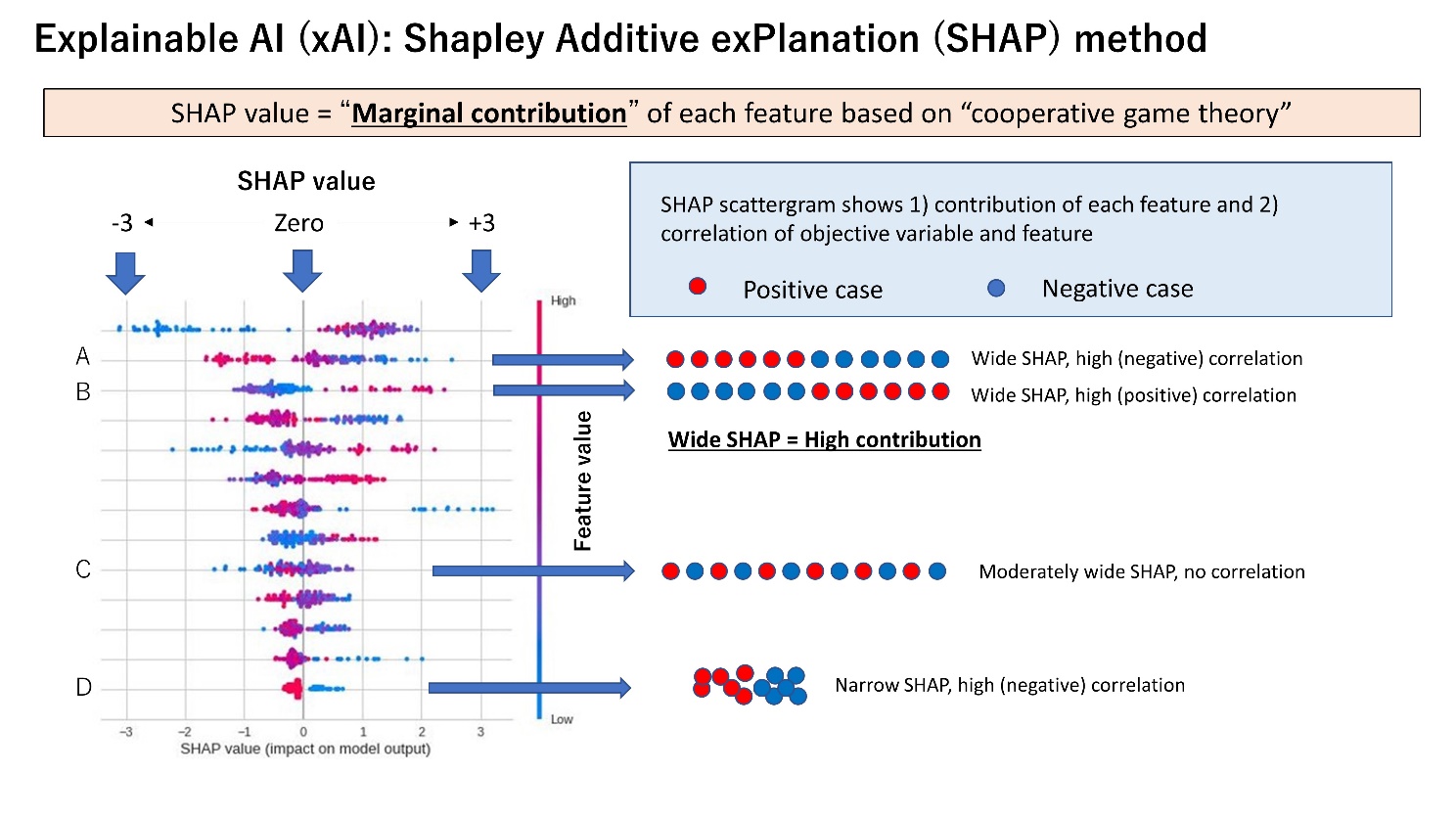
**

Explanation of SHapley Additive exPlanation (SHAP) method. SHAP method is a procedure of explain feature values in an ensemble learning, which based on “cooperative game theory”. In a game, when each player distributes to it solitarily or cooperatively, a total reward is calculated based on each coalition of all players. The Shapley value is importance of each player to the overall cooperation, which is an expected payoff.

SHAP scattergram of ensemble learning indicates two characteristics of features, contribution of each feature and correlation of objective variable and feature. In the figure, wide SHAP (feature A/B/C) indicates high contribution. Feature A and C demonstrates negative correlation, and B positive correlation.

**Supplemental file 3**

|  | Total  (N=112) | Prediction data (N=90) | Test data (N=22) | P value |
| --- | --- | --- | --- | --- |
| TTS (N, %) | 56 (50%) | 45 (50%) | 11 (50%) | N/A |
| Age (year) | 77 [67, 84] | 78 [69, 86] | 71 [60, 84] | 0.115 |
| Man (N, %) | 32 (29%) | 28 (31%) | 4 (18%) | 0.297 |
| HTN (N, %) | 42 (38%) | 33 (37%) | 9 (41%) | 0.807 |
| HL (N, %) | 50 (45%) | 38 (42%) | 12 (55%) | 0.344 |
| DM (N, %) | 24 (21%) | 16 (18%) | 8 (36%) | 0.080 |
| CKD (N, %) | 25 (22%) | 22 (24%) | 3 (14%) | 0.259 |
| BNP (pg/mL) | 199 [41, 485] | 199 [65, 484] | 216 [34, 511] | 0.940 |
| WBC (/mm^3^) | 8600 [6700, 11300] | 8700 [6800, 11400] | 7650 [6250, 10200] | 0.285 |
| CRP (mg/dL) | 0.48 [0.10, 2.63] | 0.55 [0.10, 4.64] | 0.26 [0.05, 0.79] | 0.124 |
|  |  |  |  |  |
| HR (bpm) | 87 [75, 105] | 87 [73, 107] | 87 [79, 94] | 0.823 |
| P axis (degree) | 55 [37, 69] | 56 [38, 70] | 54 [27, 66] | 0.506 |
| PR (msec) | 170 [156, 188] | 169 [156, 188] | 174 [162, 188] | 0.491 |
| QRS axis (degree) | 26 [-7, 62] | 27 [-15, 65] | 16 [11, 49] | 0.953 |
| QRS width (msec) | 90 [82, 101] | 92 [84, 102] | 89 [79, 100] | 0.224 |
| QTc (msec) | 430 [408, 449] | 430 [408, 448] | 431 [409, 468] | 0.410 |
| T axis (degree) | 69 [36, 107] | 68 [33, 104] | 72 [47, 128] | 0.323 |
|  |  |  |  |  |
| I STJ | 5 [-30, 35] | 5 [-29, 35] | -3 [-41, 36] | 0.468 |
| I STmid | 8 [-30, 41] | 10 [-24, 44] | -15 [-58, 39] | 0.272 |
| I STend | 10 [-25, 56] | 13 [-19, 55] | -15 [-58, 63] | 0.228 |
| I T | 65 [-50, 155] | 65 [-46, 151] | 65 [-85, 170] | 0.839 |
| II STJ | 18 [-35, 45] | 13 [-35, 44] | 25 [1, 46] | 0.524 |
| II STmid | 18 [-20, 56] | 18 [-24, 49] | 20 [-8, 69] | 0.500 |
| II STend | 28 [-15, 81] | 25 [-15, 80] | 33 [-5, 95] | 0.624 |
| II T | 158 [68, 274] | 158 [80, 275] | 18 [30, 245] | 0.762 |
| III STJ | 10 [-41, 55] | 5 [-49, 55] | 15 [-13, 48] | 0.444 |
| III STmid | 15 [-40, 60] | 5 [-40, 50] | 43 [4, 65] | 0.060 |
| III STend | 25 [-26, 71] | 15 [-30, 70] | 43 [11, 75] | 0.127 |
| III T | 100 [-75, 200] | 100 [-85, 200] | 93 [14, 181] | 0.794 |
| aVR STJ | -5 [-34, 25] | -8 [-31, 25] | -3 [-45, 24] | 0.902 |
| aVR STmid | -13 [-50, 20] | -13 [-49, 15] | -8 [-49, 28] | 0.947 |
| aVR STend | -25 [-66, 10] | -25 [-65, 5] | -15 [-74, 23] | 0.739 |
| aVR T | -115 [-198, -50] | -115 [-193, -65] | -105 [-215, 70] | 0.541 |
| aVL STJ | 0 [-30, 30] | -5 [-30, 44] | 0 [-24, 15] | 0.378 |
| aVL STmid | -5 [-35, 26] | 0 [-30, 30] | -10 [-53, 0] | 0.068 |
| aVL STend | -3 [-41, 35] | 0 [-40, 39] | -13 [-55, 4] | 0.091 |
| aVL T | -33 [-95, 93] | 30 [-95, 105] | -55 [-78, 50] | 0.338 |
| aVF STJ | 10 [-35, 50] | 3 [-39, 54] | 10 [5, 48] | 0.393 |
| aVF STmid | 10 [-20, 55] | 8 [-20, 59] | 33 [-4, 49] | 0.222 |
| aVF STend | 20 [-20, 71] | 15 [-25, 74] | 38 [5, 64] | 0.300 |
| aVF T | 135 [41, 224] | 125 [39, 221] | 155 [95, 261] | 0.510 |
| V1 STJ | 30 [0, 75] | 35 [0, 85] | 28 [-5, 60] | 0.250 |
| V1 STmid | 48 [15, 105] | 45 [11, 119] | 60 [21, 94] | 0.578 |
| V1 STend | 53 [14, 125] | 50 [16, 135] | 63 [11, 99] | 0.464 |
| V1 T | 95 [-45, 206] | 95 [-53, 215] | 110 [-80, 155] | 0.447 |
| V2 STJ | 110 [40, 190] | 115 [40, 215] | 90 [44, 166] | 0.403 |
| V2 STmid | 163 [93, 290] | 173 [88, 299] | 160 [100, 205] | 0.442 |
| V2 STend | 223 [100, 370] | 228 [106, 400] | 185 [100, 288] | 0.249 |
| V2 T | 345 [184, 598] | 343 [171, 671] | 353 [228, 445] | 0.631 |
| V3 STJ | 98 [-6, 206] | 105 [-5, 208] | 93 [-9, 174] | 0.692 |
| V3 STmid | 170 [79, 271] | 170 [46, 305] | 153 [56, 240] | 0.750 |
| V3 STend | 215 [64, 398] | 225 [61, 433] | 170 [65, 299] | 0.634 |
| V3 T | 380 [91, 571] | 383 [95, 601] | 355 [-61, 508] | 0.541 |
| V4 STJ | 62 [-41, 151] | 62 [-34, 148] | 55 [-85, 158] | 0.823 |
| V4 STmid | 98 [-5, 211] | 95 [-4, 210] | 108 [-13, 218] | 0.898 |
| V4 STend | 140 [15, 270] | 155 [20, 270] | 118 [15, 265] | 0.742 |
| V4 T | 250 [-145, 503] | 260 [-115, 500] | 213 [-296, 511] | 0.874 |
| V5 STJ | 15 [-36, 93] | 18 [-30, 86] | 10 [-75, 115] | 0.895 |
| V5 STmid | 35 [-31, 116] | 35 [-30, 115] | 30 [-58, 148] | 0.930 |
| V5 STend | 53 [-26, 155] | 62 [-19, 159] | 10 [-74, 118] | 0.137 |
| V5 T | 130 [-140, 275] | 113 [-130, 274] | 158 [-278, 365] | 0.829 |
| V6 STJ | 0 [-40, 44] | 0 [-40, 34] | 15 [-34, 63] | 0.457 |
| V6 STmid | 0 [-36, 58] | -5 [-34, 50] | 8 [-40, 73] | 0.468 |
| V6 STend | 15 [-35, 75] | 35 [-35, 69] | 35 [-54, 79] | 0.493 |
| V6 T | 85 [-93, 233] | 80 [-78, 219] | 130 [-250, 325] | 0.966 |

Baseline characteristics and comparison of prediction data (N = 90) and test data (N = 22). Numeric variables are displayed as median (interquartile range: 25%, 75%), and the Mann–Whitney U test was performed. Categorical variables are presented as numbers (%), and Fisher’s exact tests were performed. ST J/ STmid/ STend, and T wave are expressed as μV, and are shown in Figure 2. Other abbreviations include the following: TTS, Takotsubo syndrome; HTN, hypertension; HL, hyperlipidemia; DM, diabetes mellitus; CKD, chronic kidney disease; BNP, brain natriuretic peptide; WBC, white blood cell; CRP, C-reactive protein; HR, heart rate; N/A, not applicable. Statistical significance was set at P < 0.05.

**Supplemental file 4**

**
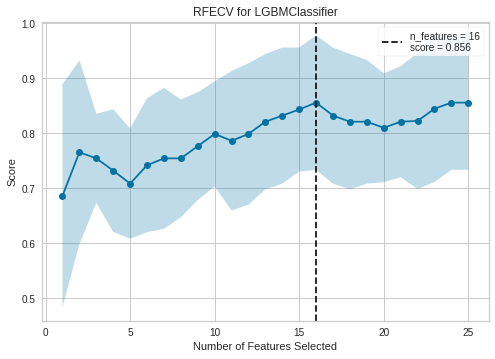

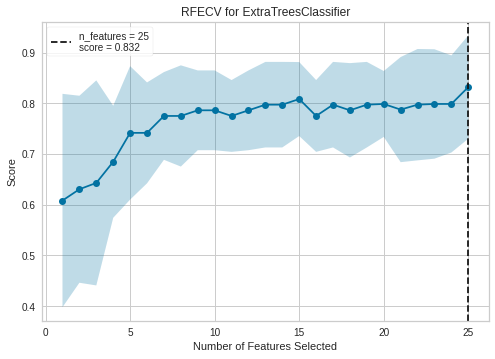
**

Results of searching the best number of features in model_LGBM and model_ET by recursive feature elimination by cross validation (RFECV) on PyCaret, which fits a model and removes the weakest features until the specified number of features is found. By recursive elimination of a small number of features per loop, RFE tries to eliminate dependencies and collinearity that may exist in the model.

Model_ET shows the best number of features is 25, and model_LGBM 16, 24, and 25. As the results, we adopted all 25 features to construct the ML models.

**Supplemental file 5.**

**
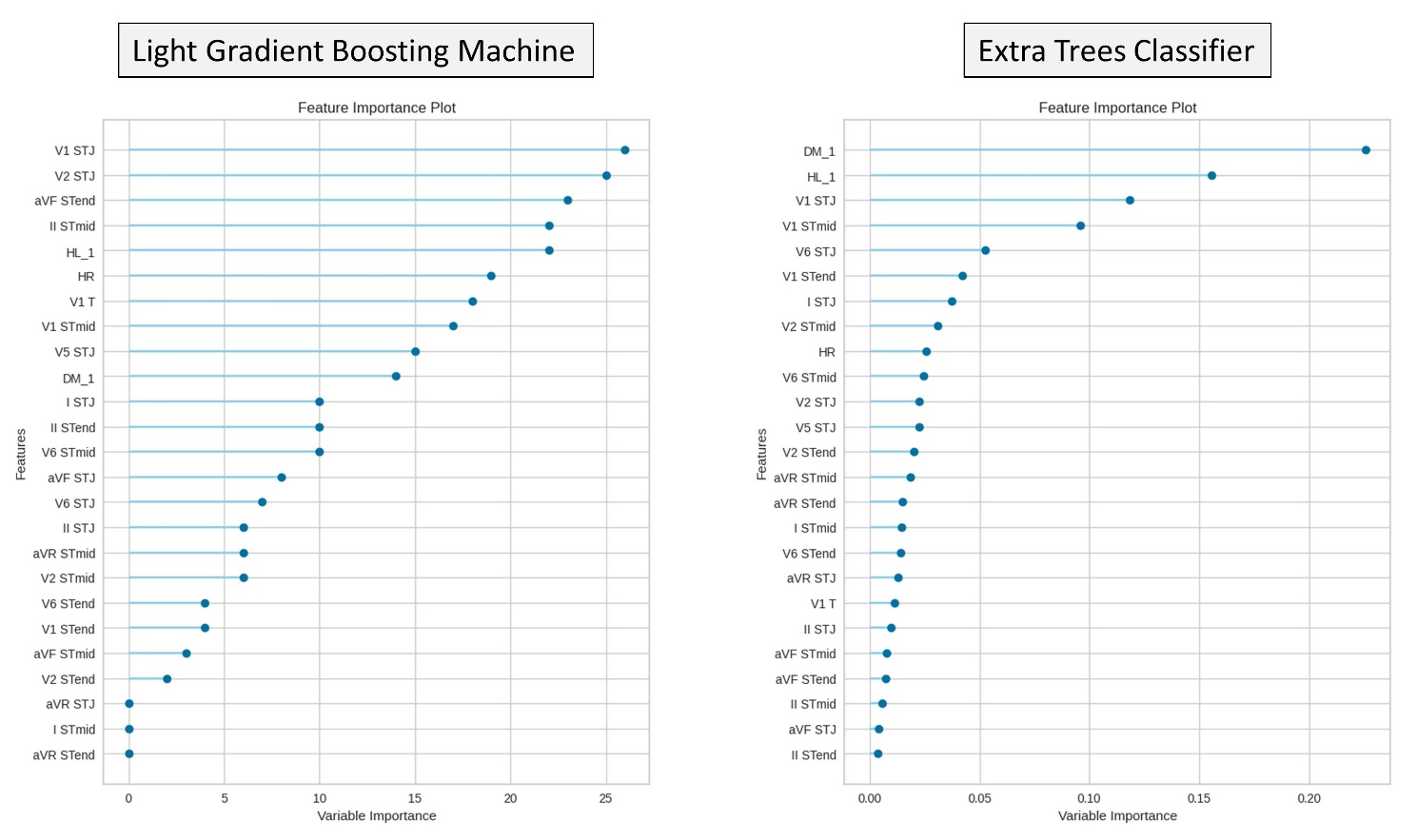
**

Feature importance plot of two machine learning models. Left side: Plot of Light Gradient Boosting Machine (model_LGBM), Right side: Plot of Extra Trees Classifier (model_ET). X-axis of model_LGBM figure shows frequency of usage in the model, and of model_ET shows percentage of importance whose summation is 1.00, and both show average of 10-fold train procedures. Features are arranged in the order of importance on the Y-axis. Abbreviations are explained in legends of footnote of Table 1.

**Supplemental file 6.**

Hyperparameters in finalized models

<model_ET>

bootstrap=False, ccp_alpha=0.0,

class_weight=None, criterion='gini',

max_depth=None, max_features='auto',

max_leaf_nodes=None, max_samples=None,

min_impurity_decrease=0.0,

min_impurity_split=None,

min_samples_leaf=1, min_samples_split=2,

min_weight_fraction_leaf=0.0,

n_estimators=100, n_jobs=-1,

oob_score=False, random_state=34,

verbose=0, warm_start=False

<model_LGBM>

boosting_type='gbdt', class_weight=None,

colsample_bytree=1.0, importance_type='split',

learning_rate=0.1, max_depth=-1,

min_child_samples=20, min_child_weight=0.001,

min_split_gain=0.0, n_estimators=100, n_jobs=-1,

num_leaves=31, objective=None, random_state=34,

reg_alpha=0.0, reg_lambda=0.0, silent='warn',

subsample=1.0, subsample_for_bin=200000,

subsample_freq=0
